# Utilization of partograph and its associated factors among obstetric caregivers in public health institutions of Southwest Ethiopia

**DOI:** 10.1186/s12884-021-03822-5

**Published:** 2021-05-27

**Authors:** Abel Girma Tilahun, Dawit Getachew Gebeyehu, Yayehyirad Yemaneh Adinew, Fekede Woldekidan Mengstu

**Affiliations:** 1 Department of Public Health, Mizan Tepi University College of Health Sciences, Mizan Teferi, Ethiopia; 2 Department of Midwifery, Mizan Tepi University College of Health Sciences, Mizan Teferi, Ethiopia

**Keywords:** Utilization, Partograph, Southwest Ethiopia

## Abstract

**Background:**

Partographs should be used universally to monitor the mother and fetus’s conditions during delivery. However, its application in different parts of the world, including Ethiopia, is inconsistent. Moreover, its magnitude has not been determined in study area. As a result, the aim of this study was to investigate the utilization of partograph and associated factors among obstetric caregivers in public health institutions of Southwest Ethiopian.

**Methods:**

An institutional-based cross-sectional study was conducted in Southwest Ethiopia from March 1st to June 30th, 2018. A simple random sampling technique was used to select study participants. A self-administered questionnaire was used to gather data on background characteristics, knowledge of partograph, and partograph utilization. The collected data were entered into an EPI Info and analysed using SPSS Version 22. We used bivariate and multivariate logistic regression analysis. Frequencies, tables, and graphs were used to present the final results. To determine statistical significance, a *P*-value of less than 0.05 was used.

**Result:**

The response rate of this study was 393(92.2 %). The magnitude of utilization of partograph was 43 % with (95 % CI: 38.4, 48.1). According to the multivariate analysis being nurse or health officer [AOR = 0.37(0.21, 0.66)], degree level educational qualification [AOR = 0.32 (0.17, 0.60)], being trainined on partograph [Adjusted OR = 7.83 (95 % CI: (4.54, 13.50)], good knowledge about partograph [AOR = 5.84 (95 % CI: (3.27, 10.44)] and working at health center [AOR = 1.99 (95 % CI: (1.12, 3.52)] were found as determinants of partograph utilization.

**Conclusions:**

The magnitude of partograph utilization among obstetric caregivers was found to be low in this study. Partograph utilization was determined by the type of profession, qualification level, knowledge of partograph, in-service training, and type of institution. To ensure its regular, obstetric caregivers must receive training and gain knowledge about it.

**Supplementary Information:**

The online version contains supplementary material available at 10.1186/s12884-021-03822-5.

## Background

About 295,000 women died in 2017 as a result of a preventable cause of maternal mortality (MM). The majority of these deaths (94 %) occurred in low and middle-income countries; Subsaharan Africa (SSA) and Southeast Asia (SEA) combined accounted for 86 % of these deaths, and SSA alone accounting for nearly 66 %. Although the maternal mortality rate (MMR) has declined by 60 % in SEA from 2000 to 2017, in SSR, it was reduced by 40 % only [1, 2]. According to the 2016 Ethiopian Demographic and Health Survey (EDHS), Ethiopia’s MMR was 412/100,000 live births, with a Neonatal Mortality rate of about 30 deaths per 1000 live births [3].

Maternal death may occur at any time during pregnancy, childbirth, or the postpartum period. MM may be caused by pre-eclampsia and eclampsia during pregnancy, labour complications, bleeding during and during delivery, infection after childbirth, and abortion [1, 4].

To reduce the catastrophe of maternal death, feasible intervention are available such as the utilization of partograph. Partograph is a preprinted paper, which is used to monitor the progress of labor and the change observed on mother and fetus during labor. It was first used in 1950; its use became an international standard method in 1987 in Nairobi, Kenya, and in 1994, the World Health Organization declared its essential use in all seating for enhancing labor management and lowering maternal and foetal mortality [1, 2]. It was, also demonstrated that in labor monitored by parthograph prolonged labour was reduced from 6.4 to 3.4 %, augmentation was reduced from 20.7 to 9.1 %, emergency caesarean section was reduced from 9.9 to 8.3 %, and stillbirths were reduced from 0.5 to 0.3 % [2].

Despite the WHO recommendations, the utilization of partograph was not consistent in every setting. For example, in Bangladesh Partographs were used for 98 % of women in labor [5]. But, in Africa the magnitude of partograph utilization was less than 50 % [6–8].

According to different studies done in Ethiopia the magnitude of the utilization of partograph shown to have a wide variation from region to region and across zones of the same region [9–18]. Studies from the Central and Eastern zone of Tigray regional state reported the highest level of utilization from around 70 to 83 % [10, 13]. While a study from the west shoa zone of Oromiya regional state reported the lowest utilization of partograph which was 31 % [9]. The magnitude of utilization of partograph in others region such as East Gojam Amhara Region, was 53 % [19], in SNNPRE Hadiya zone 54 %, and Wolayita Zone 71 % [12, 17]. In Addis Ababa, 57 to 69 % of obstetric caregivers utilized partograph routinely [11, 16]. The largest diference in the utilization of partograph is seen in different zones of Oromia region ranging from 31, to 71 % [9].

Regarding the determinants of partograph utilization, studies had showing an association with different variable. These variable were socio-demographic characteristics of obstetric caregivers (age, gender, profession, qualification, service year, and types of health institution), knowledge regarding partograph and receiving training regarding partograph [5–21].

Eventhough, similar studies were performed in various parts of the world, including Ethiopia, the results were not generalizable due to geographic and socioeconomic heterogeneity, and also such information was lacking in southwest Ethiopia. Therefore, this study investigated the magnitude of utilization of partograph among obstetric care givers in public health institutions of southwest Ethiopia.

## Methods

### Study setting and period

The research was carried out in public health institutions in four zones in South West Ethiopia: Bench-Shako, Kafa, West-omo, and Shaka. These areas had a total population of about 3,291,083. Regarding the health infrastructure in the study area there are six hospitals (1 university teaching hospital, two zonal hospitals, and three primary level hospitals) and over 80 health centres. This study was condacted from March 1st to June 30th, 2018.

### Study design and population

 Institution based cross-sectional study design was conducted to assess the magnitude of partograph utilization and associated factors among obsetetrci care givers. The source population of this study were all health professionals who were working in the obstetric unit of public health institutions of Kaffa, Bench-shako, West-Omo and Sheka Zones. The study population were all randomly selected health professionals who were working in the randomly selected public health institutions of Kaffa, Bench-shako, West-Omo and sheka zones.

### Inclusion and exclusion criteria

The study included health professionals who had worked in the obstetric ward for the last six months, such as nurses, midwives, and health officers. The study excluded professionals who did not work in the obstetric ward for less than six months.

### Sample size determination

The sample size was calculated using the single population proportion formula, with a 95 % confidence interval (CI), a 5 % margin of error, and a 50 % utilization rate of partograph. The calculated sample size was 384, and the final sample size was 423 after accounting for the 10 % non-response rate.

$$ n=\frac{{\underset{\overline{2}}{\left({\mathrm{Z}}_{\partial}\right)}}^2\mathrm{P}\left(1\hbox{-} \mathrm{P}\right)}{{\mathrm{d}}^2}\kern2em n=\frac{(1.96)^20.5\left(1-0.5\right)}{(0.05)^2} $$

### Sampling procedure

The study participants were selected by using simple random sampling method, after proportional allocation of the sample size to each randomly selected institution based on the number of caregivers working in the health institution.

### Data collection procedures and quality control

To collect the data a structured questionnaire was used, which was adopted from similar work [6, 20, 21]. The questionnaire has three parts the first part was about the socio-demographic characteristics of the participants including age, sex, profession, qualification, and experience. The second part was about the utilization of partograph and the third part was about other determinants of partograph utilization such as training regarding partograph, knowledge regarding partograph and types of public health institution where obstetric caregivers work. To ensure the data quality the questionnaire was pre-tested, the data collectors were given training on the objective and purpose of the study and all stages of the data collection were supervised.

### Data processing and analysis

The data were entered into Epi Info Version 3.5.1 and double-checked for accuracy, and transported to the statistical software package for social science (SPSS) version 22 for analysis. Bivariate and a multivariate logistic regression was performed to identify factors that are associated with utilization of partograph. Finally, the adjusted odds ratio (AOR) and its 95 % confidence interval (CI) were reported. A cut-off point for statistical significance is a *P*-value of less than 0.05.

## Result

### Socio-demographic charachterestics of the participant

The study had a 92.9 % response rate. The average age of study participants was 22.5 years old, with 46.4 % of them being female. In terms of profession and qualification, 173 (44 %) of the participants were Midwives, while 224 (57 %) expected to hold a diploma. The majority of respondents, 206 (52.4 %), have five or less years of experience (Table 1).

**Table 1:** Socio-Demographic Characteristics of the Respondents on Utilization of Partograph Southwest Ethiopia 2018. *N* = 393

Variable	Category	Frequency	Percent (%)
**Age**	> 30	220	56.0
	< = 30	173	44.0
**Sex**	Male	210	53.4
	Female	183	46.6
**Profession**	Midwife	173	44.0
	Others	220	56.0
**Qualification**	Diploma	224	57.0
	Degree	169	43.0
**Experience**	≤ 5 years	206	52.4
	> 5 Years	187	47.6

### Utilization of partograph among obstetric caregivers

**Fig. 1:**
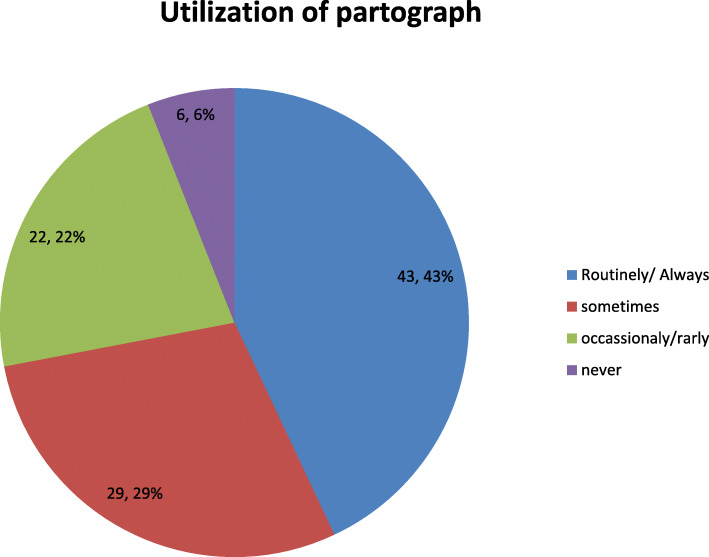
Utilization of partograph among obstetric caregivers in public health institution of Southwest Ethiopia, 2018

In this study, the magnitude of partograph utilization was 43 % (95 % CI: 38.4, 48.1). This means 169(43)% of obstetric caregivers utilized partograph routinely, while 113(29 %) of the participant utilized it sometimes and 87 (22 %) utilized partograph occasionally (Fig. 1).

### Reasons for not using the partograph

**Fig. 2:**
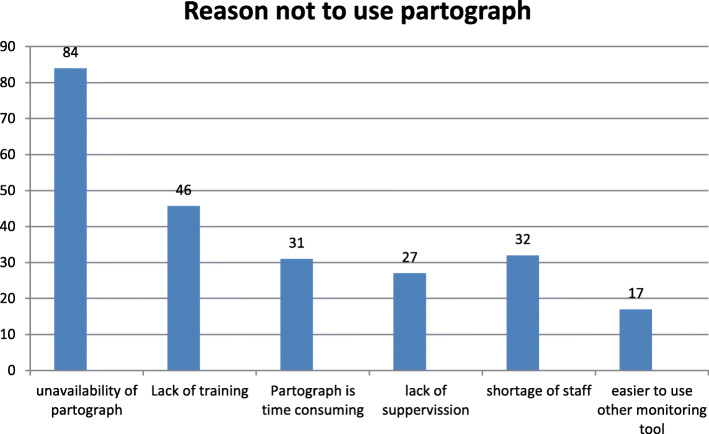
Reasons for not using partograph among Obstetric caregivers in Southwest Ethiopia, 2018

Unavailability of partograph 84(37.5 %), lack of training on how to use partograph 46(20.53 %), partograph is time consuming 27(12.05), shortage of staff 32(14.28 %) and it is easier to use another monitoring tool 17(7.58 %), were the reasons given for not utilizening partograph routinely in the study area (Fig. 2).

### Factors associated with partograph utilization

In bivariate analysis, variables such as age, gender, profession, qualification, previous training, knowledge regarding partograph and types of the institution were associated with utilization of partograph at *p*-value lessthan 0.2. In Multiple variable regression, profession, qualification, previous training, knowledge regarding the partograph, and the types of health institution were significantly associated with the utilization of partograph at *P*-value less than 0.05 (Table 2).

In this study, being a Nurse or Health officer decrease utilization of partograph by 63 % as compared to being Midwifery professionals. [Adjusted OR = 0.37(0.21, 0.66)]. Similarly, being a degree holder in qualification decrease partograph utilization by 68 % as compared to diploma level obstetric care giver. [Adjusted OR = 0.32 (0.17, 0.60)]. Also, the odds of partograph utilization for obstetric caregivers who received on job training was 7 times higher than those not got training [Adjusted OR = 7.83 (95 % CI: (4.54, 13.50)]. In addition, the odds of partograph utilization was 5.84 times higher among obstetric caregivers who have good knowledge of partograph as compared to those who have poor knowledge of partograph [Adjusted OR = 5.84 (95 % CI: (3.27, 10.44)]. Finally, the odds of partograph utilization was 1.99 times higher among caregivers who works in hospital as compared to caregivers who works at health centers. [Adjusted OR = 1.99 (95 % CI: (1.12, 3.52)] (Table 2).

**Table 2:** Multiple variable logistic regression analysis of the utilization of partograph among obstetric caregivers in Southwest Ethiopia 2018. (*N* = 393)

Characteristics	Not utilized N(%)		Utilized N(%)	Crude OR(95 % CI)	Adjusted OR(95 % CI)
**Age**					
Below 30	117 (55.7)	93(44.3)		1	
Above 30	107 (58.5)	76(41.5)		0.89 (0.60, 1.67)	
**Sex**					
Male	118 (57.3)	88(42.7)		1	
Female	106 (56.7)	81(43.3)		1.03( 0.69, 1.52)	
**Profession**					
Midwife	92 (53.2)	81(46.8)		1	1
Others	132 (60.0)	88(40.0)		0.76 (0.51, 1.13)	0.37 (0.21, 0.66) **
**Qualification**					
Diploma	115 (51.3)	109(48.7)		1	1
Degree	109 (64.5)	60(35.5)		0.58 (0.38, 0.88 )	0.32(0.17, 0.60)
**Previous training**					
no	160 (74.1)	56(25.9)		1	1
yes	64 (36.2)	113(63.8)		5.04(3.28, 7.77)	7.83 (4.54, 13.50) **
**Knowledge**					
poor knowledge	140 (70.0)	60(30.0)		1	1
good knowledge	84 (43.5)	109(56.5)		3.02( 1.99, 4.55)	5.84 (3.27, 10.44)**
**Type of institution**					
Hospital	89 (49.7)	90(50.3)		1	1
Health center	135 (63.1)	79(36.9)		1.75(1.15, 2.58)	1.99 (1.12, 3.52) **

Note: * *p* value < 0.2, ** *p* value < 0.05

## Discussion

### Level of partograph utilization

In this study, the magnitude of partograph utilization was 43 % (95 % CI: 38.4, 48.1). This is inline with similar study findings done in North Shoa and Addis Abeb, Ethiopa [15, 19]. But it is lower than simiar study conducted in Tigray, Oromia and SNNPRE [10–14, 16]. However, this finding was higher than a similar study conducted in cameron, Nigeria and West Shoa zone of oromia [6, 8, 9]. These differences could be due to the socio-political and economic differences of the studied area and also due to professional differences as indicated in some studies, only midwifery caregivers were studied [7, 11].

### Factor associated with utilization of partograph

In the multiple variable logistic regression model non of the socio-demographic factors, showed a significant association with utilization of partograph among obstetric caregivers. This evidence was supported by other studies [9, 15, 16, 19]. But, in another study a significant association was showen between sociodemographic factors and utilization of partograph [10, 13, 14].

In this study, being a nurse or a health officer decreased partograph utilization by 63 % when compared to being a midwife. Similar studies conducted in Ethiopia and elsewhere supported the findings. [13, 14, 18]. Midwives may receive more training on how to use a partograph than a nurse or health officer, and midwives may remain in the obstetric ward while the nurse or health officer rotates from ward to ward. As a result, midwives develop the skill and habit of using partographs.

Being a degree holder decreases, partograph utilization by 68 % compared to diploma holder obstetric caregiver. This finding is in contrast to similar research finding [13, 18, 19]. Degree-holding obstetric caregivers prefer to monitor the progress of labor with a piece of paper, but diploma-level caregivers are subject to strict supervision and control, so they could have used partographs more frequently to monitor women in labor.

The odds of using Partograph were seven times higher among obstetric caregivers who received on-the-job training than among those who did not. A similar study substantiates this finding [9–15, 19]. Because training refreshs and stimulates caregivers on the importance of partograph utilization; it incrases the utilization of partograph.

In addition, the odd of partograph utilization was 5.84 times higher among obstetric care givers who have good knowledge of partograph as compared to who have poor knowledge. This finding was consistent with similar previous studies [11, 13, 19]. The good knowledge of partograph is a pre request for regular utilization.

Finally, the odds of using a pantograph were 1.99 times higher among caregivers working in health centres versus those working in hospitals. The reason could be that in the hospital, emergency obstetric care is readily available, so caregivers can refer or call for an obstetrician, allowing them to focus less on filling out the partograph.

## Conclusion and recommendations

The utilization of a Partograph among obstetric caregivers in the study area is low. Being a nurse or health officer and being a degree level qualifications were negatively associated partograph utilization, while receiving training, good Knowledge regarding the partograph and working in health center types of institution were positively associated with it. Utilization of partograph can be improved by assigning midwives in the delivery ward, offering on-the-job training, and improving the knowledge of obstetric caregivers. Inaddition, qualitative study my help to dig out the barriers of partograph utilization among obstetric caregivers who have higher qualification and those who were woring in hospital level institutions.

## Supplementary Information

**Additional file 1.** Partograph data set. The data set which was usedto conclude this article

## Data Availability

All data generated or analysed during this study are included in this published article and its supplementary information files (Additional file 1).

## Abbreviations

AOR: Adjusted odds ratio
CI: Confidence interval
EDHS: Ethiopian Demographic and Health Survey
MM: Maternal mortality
MMR: Maternal Mortality Rate
SNNPRE: South Nations Nationalities and Peoples Region of Ethiopia
SPSS: Statistical software package for social science
SSA: Subsaharan Africa
SEA: Southeast Asia
WHO: World Health Organization
